# Supplementary material for: Microfluidic Chamber Design for Controlled Droplet Expansion and Coalescence
Source: Micromachines (Basel). 2020 Apr 10;11(4):394. doi: 10.3390/mi11040394 (PMC7231328; doi:10.3390/mi11040394)
Supplement: Supplementary file 1 [file micromachines-11-00394-s001.zip › micromachines-757573-supplementary-english_rev.docx]

Supplementary Materials: Microfluidic Chamber Design for Controlled Droplet Expansion and Coalescence

Mark Kielpinsk, Oliver Walther, Jialan Cao, Thomas Henkel, J. Michael Köhler and
G. Alexander Groß

The following supporting information is available:

**1. Supporting Videos**

| **micromachines-757573-VideoS1.mp4**: Droplet-formation mode (Figure 1A) |
| --- |
| 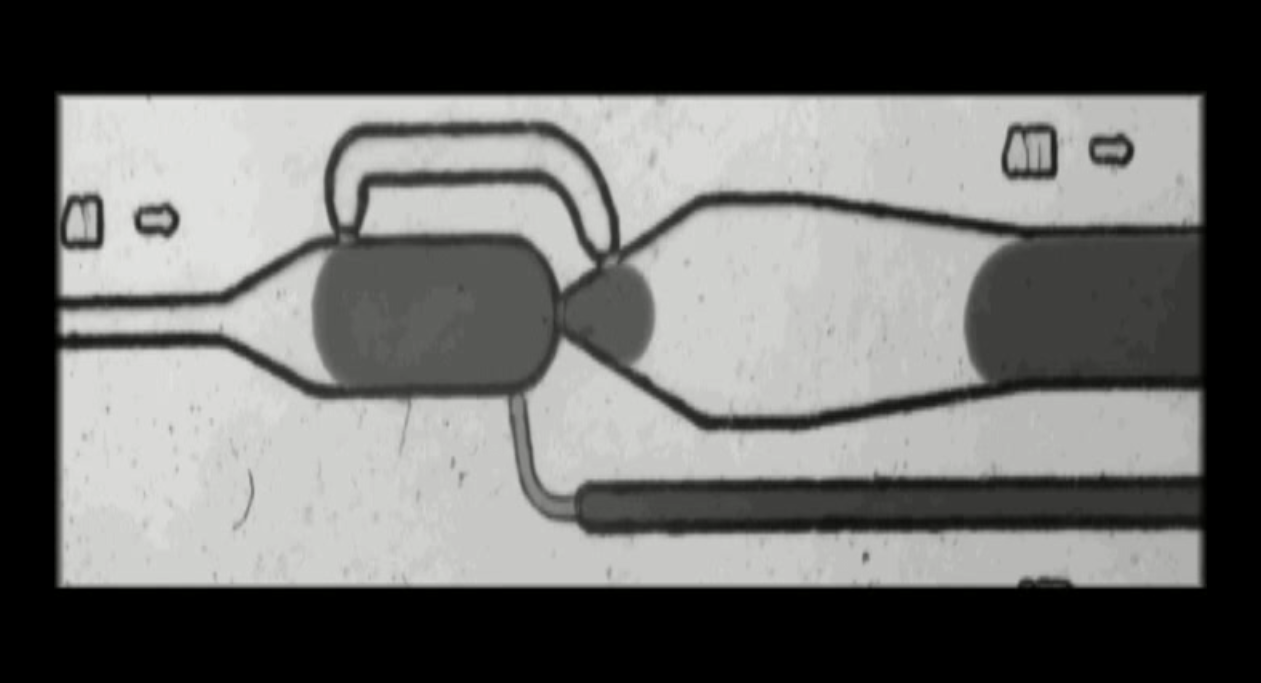 |
| The video shows the self-controlled and sequential generation of approximately 650 nL droplets. At the break-through point the droplet has a volume of about 500 nL. While passing the main-chamber nozzle the droplet was expanded by the feeding channel. The flow-rate for the carrier media and the dark dyed feeding media is 200 nL/s. |
| **micromachines-757573-VideoS2.mp4**: single drop dilution mode (Figure 1B) |
| 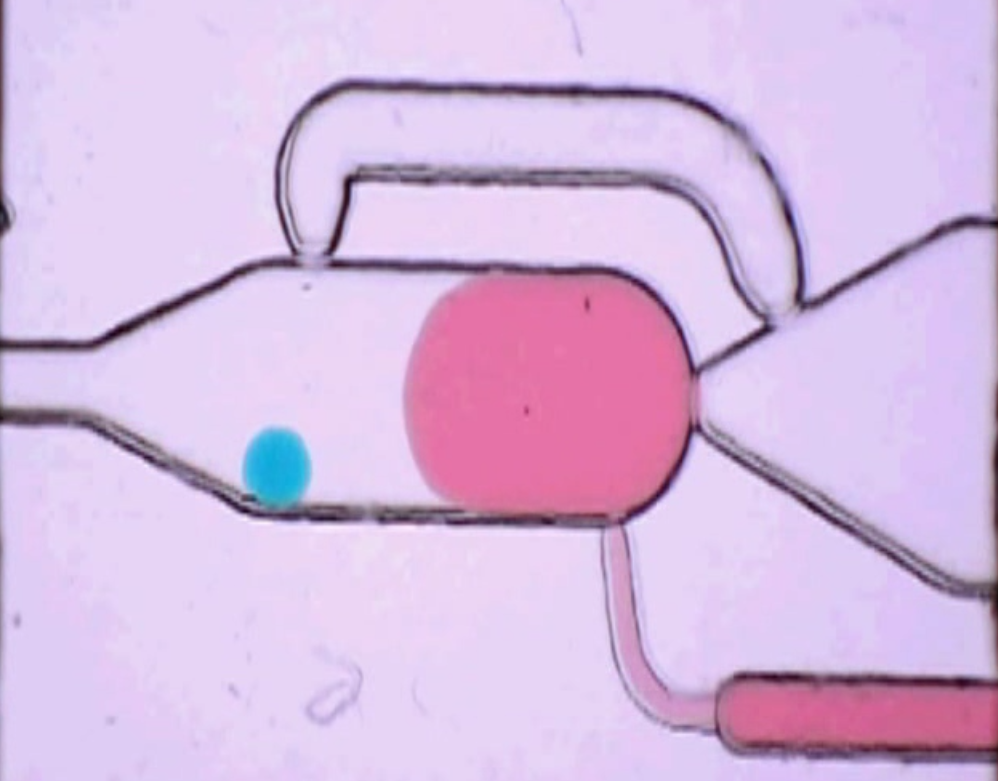 |
| The video shows the sequential coalescence of a single 40 nL droplet with a second droplet preformed in the chamber. Electro coalescence pulses were applied constantly. |
| **micromachines-757573-VideoS3.mp4**: multiple drop dilution mode (Figure 1C) |
| 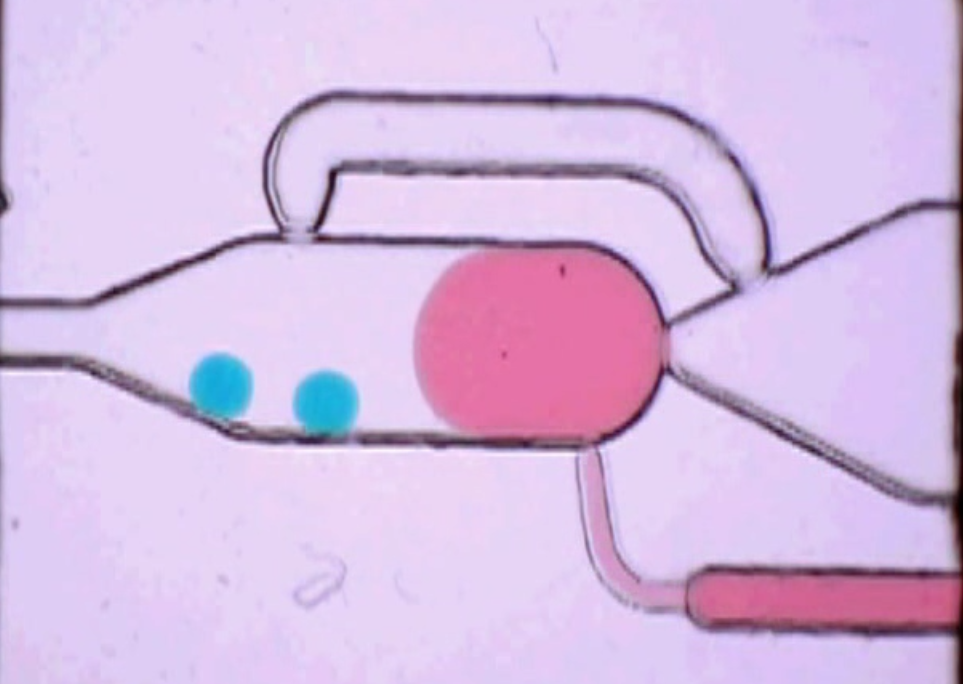 |
| The video shows the sequential coalescence of two 40 nL droplet with a preformed droplet. Electro coalescence pulses were applied constantly |
| **micromachines-757573-VideoS4.mp4**: Multiple drop coalescence mode (Figure 1D) |
| 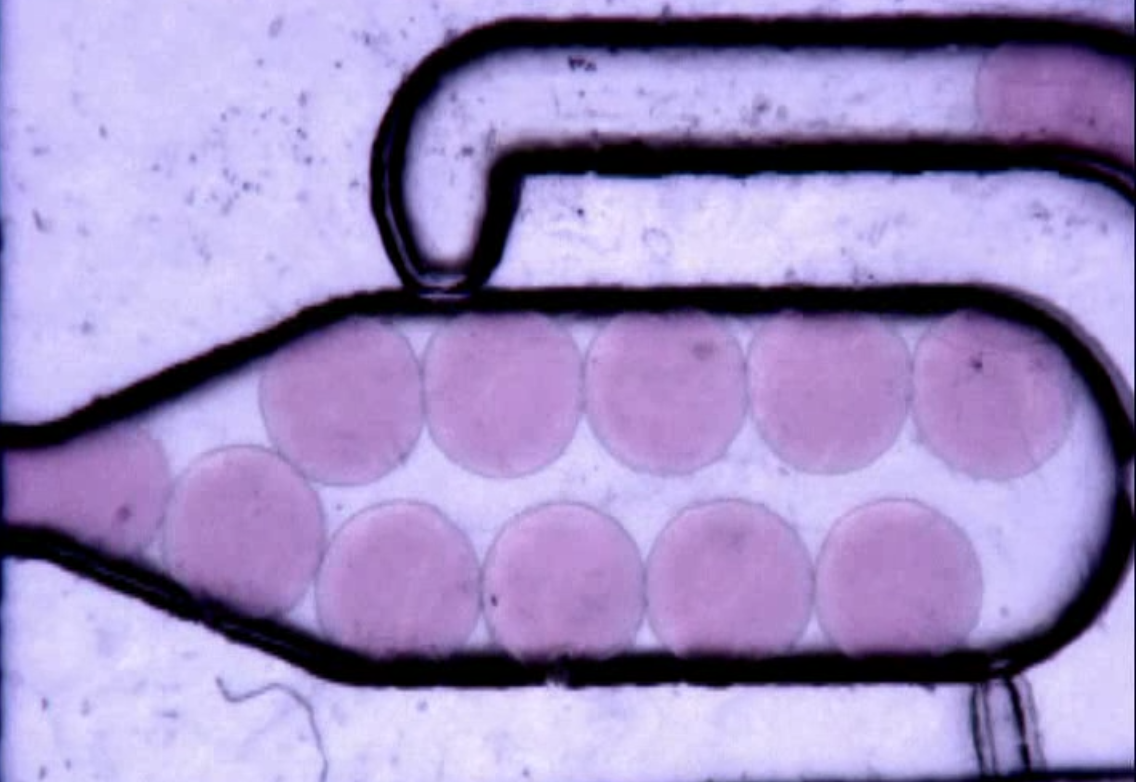 |
| The video shows the sequential coalescence of 12 individual 40 nL droplets to a unified 480 nL droplet. The electro coalescence pulses were applied in constant intervals of about 6 s regarding to the applied flow rate. |

**2. Additional Images**
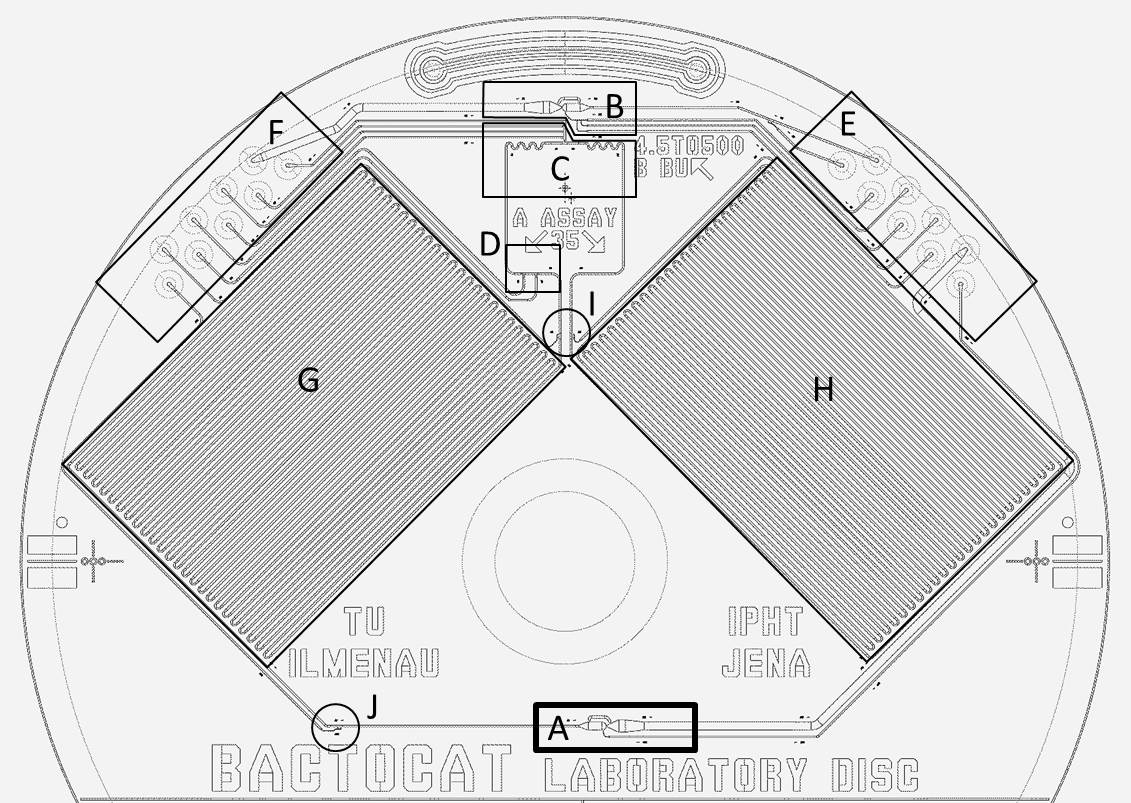


**Figure S1.** Overview of the entire fluidic network comprising operation units for approximately 35 nL and 500 nL droplets.

The fluidic network device comprised of the following operation units:

**A**: Bypass chamber. The described bypass chamber connects both network parts. Electrodes for electro coalescence are not shown.

**B**: Bypass chamber designed as 500 nL volume droplet generator.

**C**: 35 nL droplet generator and droplet mixing meanders

**D**: Double T-junction for sorting purposes

**E** and **F**: Contact area for tubing interface

**G** and **H**: Storage channels for 35 nL droplets

**I**: Droplet-distance control units

**J**: Drop-distance control unit. This unit was used to adjust the number and frequency of the 35 nL droplets which were delivered into the bypassed chamber.


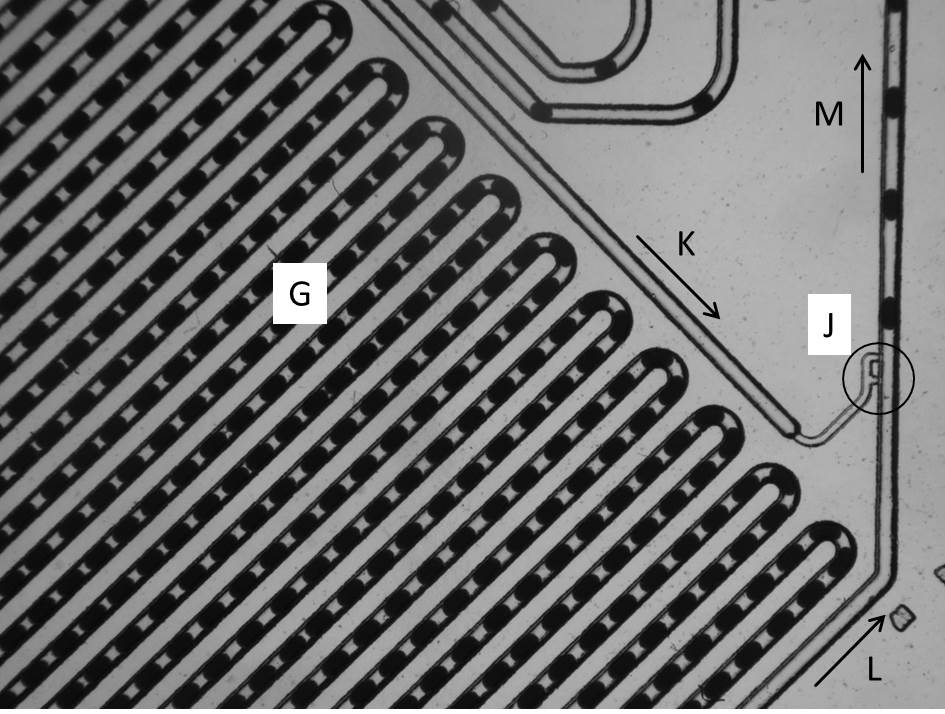


**Figure S2**. Image of the droplet storage network part. Droplet storage in network part **G** (Figure S1) with dense packing of approx. 35 nL droplets which were prepared by the droplet generator **C** (Figure S1). The drop distance control **J** was used to adjust the distance and frequency of droplets leaving the storage part along channel **L**. Drop distance- and frequency-control was realized by adjusting the flow rates of channel **L** and the carrier medium feed through channel **K**. The spaced droplet sequences in channel **M** were guided into the bypassed chamber **A** (Figure S1) for further dilution or coalescence operations.


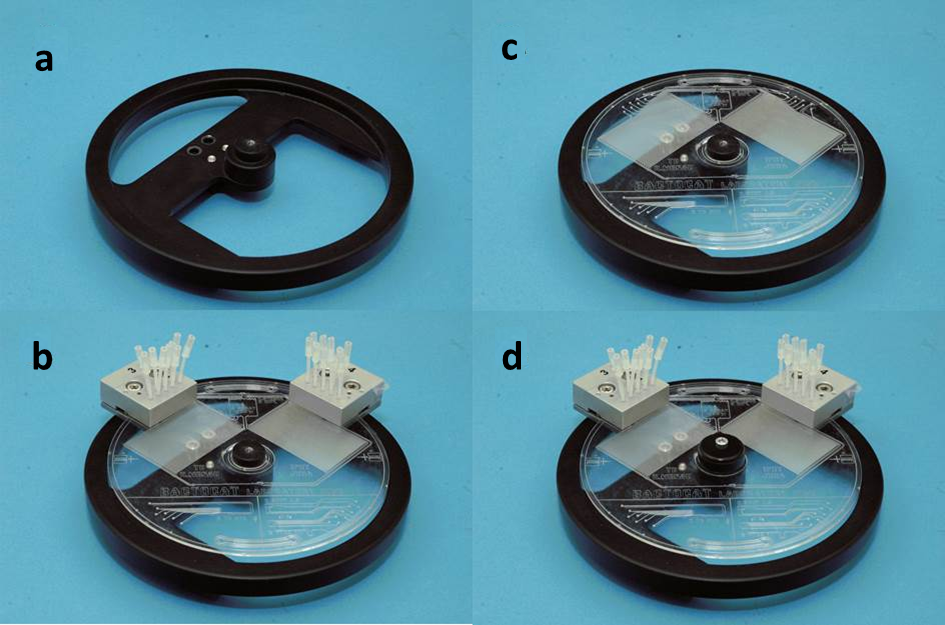


**Figure S3.** Assembling of the CD-borne fluid network in a handling chuck and fluid connector attachment.

The Bactocat-Laboratory-Disc was made of poly carbonate by application of CD (compact disk) production technology and mounted in a custom-made chuck. The assembly allows the optical monitoring of droplet processes by standard microscope or camera equipment. As fluidic interfaces, a 9-port connector was developed.


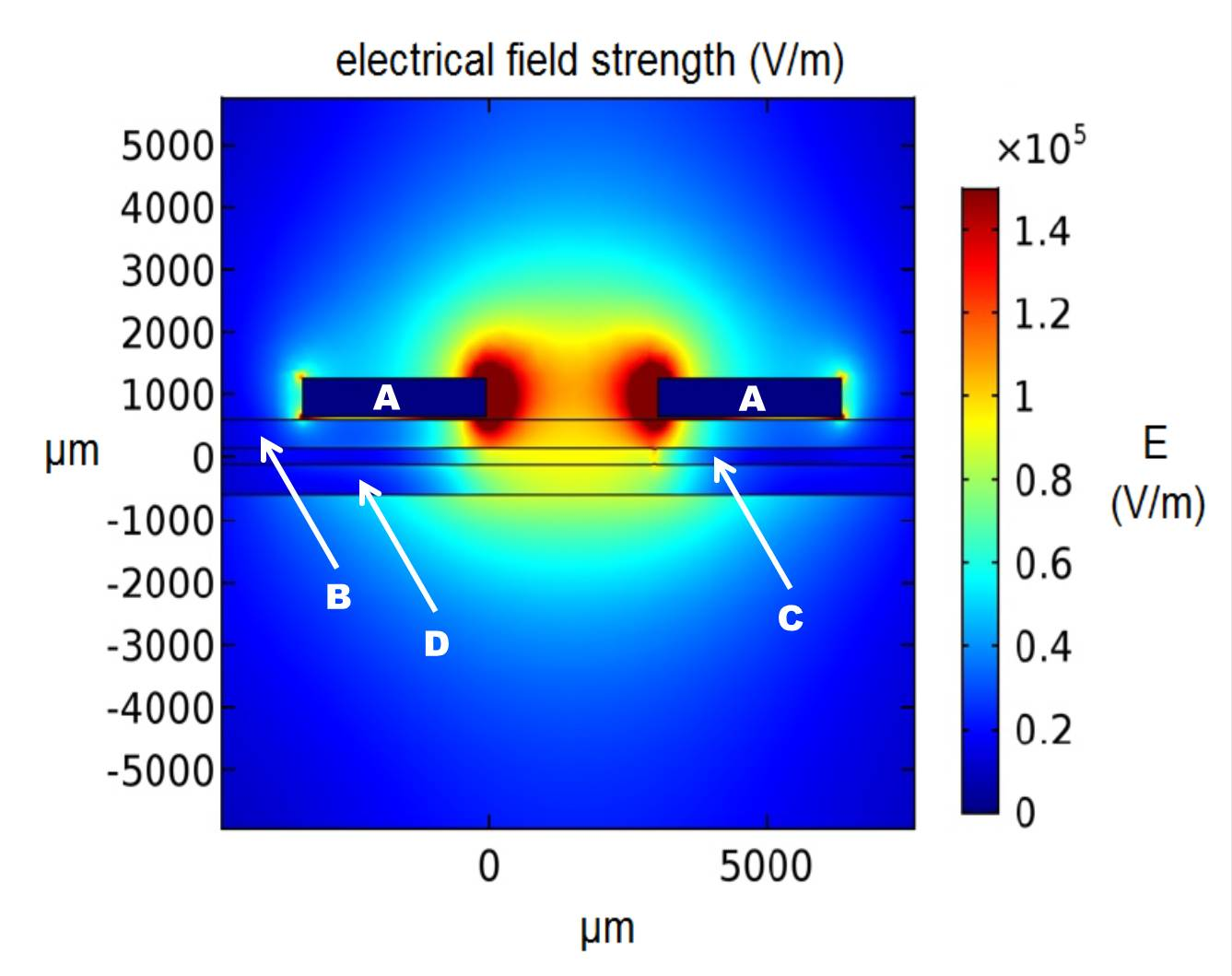


**Figure S4.** Simulation of the electrical field in the applied coalescence arrangement. (**A)** electrodes with gap between; (**B**) upper half-shape of Bactocat-Laboratory-Disc; (**C**) micro-channel with fusion chamber; (**D**) lower half-shape of Bactocat-Microfluidic-Laboratory-Disc.

The electrical field distribution was simulated by simple geometry model of the experimental arrangement. The simulation was performed using a finite element simulation tool (COMSOL Multiphysics). The symmetrical arrangement of the electrodes leads to a symmetric field distribution. The simulation shows that the highest field strengths are near the edges of the electrodes. Under typical experimental conditions (450 V total voltage), a maximum electrical field strength of about 0.15 MV/m was present near the electrode, and of about 0.11 MV/m inside the fusion chamber. The simulation gives information about the attenuation of electric-field from electrodes towards the fusion chamber which can expect. Additionally, it is shown that the field strength increases rapidly outside the electrode cap. Unintentional droplet-fusion outside the fusion-chamber should be very well controllable.

**3. Additional Experimental Results**

In some cases, the first step in the coalescence of two droplets could be observed also in the case that the droplets are not in direct contact. This is well illustrated by microscopic images of a fusion chamber immediately before and after switching on the electrical field. The normal sphere-like liquid interfaces are observed inside the fusion chamber if no electrical field was applied (Figure S5a). The laterally applied electrical field induces a deformation of the droplets in the direction of the electrical field (Figure S5b). The surfaces of both droplets are stretched one against the other until they form sharp tips. In addition, the formation of a small water bridge between both droplets was observed which seems to present the beginning of the unification of the both droplets. After application of an electrical field, the coalescence between contacting droplets in the fusion chamber takes place within 500 ms. In case of the fusion of a droplet set, this fast-primary fusion is followed by a slower shape relaxation process forming the final droplet. This process can take up to 1 s.


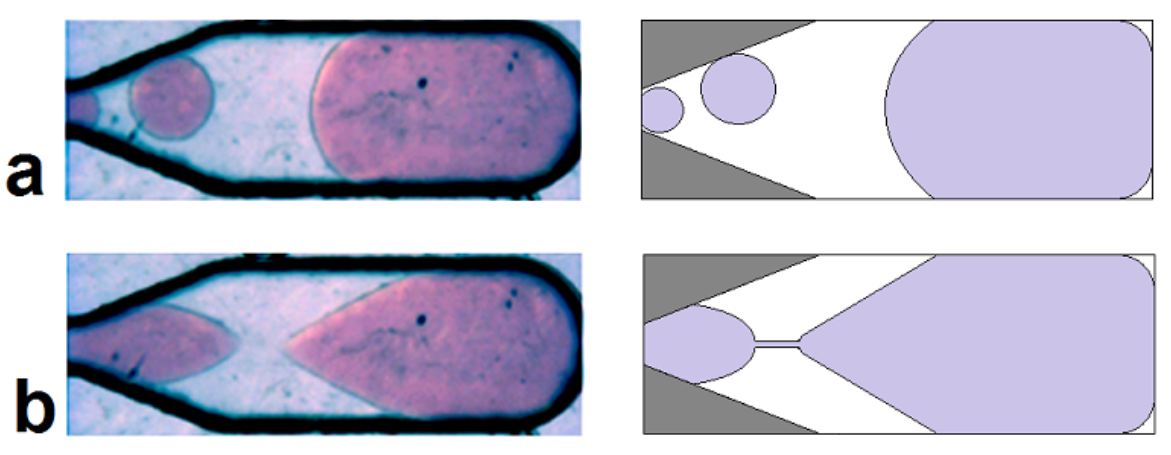


**Figure S5**: Polarization-induced droplet deformation and formation of a water bridge in the initial phase of droplet fusion: (**a**) without electric field, (**b**) after switching on electric field a deformation of droplets and formation of a very small water bridge occurs

The quality and reliability of droplet fusion efficiency was analyzed by segment distance /segment size diagram plots for the received coalesced droplets. Therefore, a photometric sensor was placed at the outlet channel and the time intervals for passing segments were detected. At a constant flow rate, the duration time of a segment is proportional to the segment volume. The duration time between two segments represents the volume of continuous-phase between the segments. The correlation between both parameters represents the sequence quality on the one hand and the droplet formation reliability on the other. In Figure S6 a received scatter plot is shown exemplary. Here, the multiple droplet coalescence-based sequence quality for highly viscous droplets (20 Vol% glycerol) for two different coalescence voltages are shown. In Figure S6a a large cloud of points—caused by many droplets with significant deviations in size and distance—a, re-formed at about 300 V. A much more homogeneous population of product droplets was obtained at 450 V, where only a view data points indicates droplets with deviating size and distance. The enhanced viscosity, received by addition of glycerol had only small effects on the coalescence probability at lower voltages.


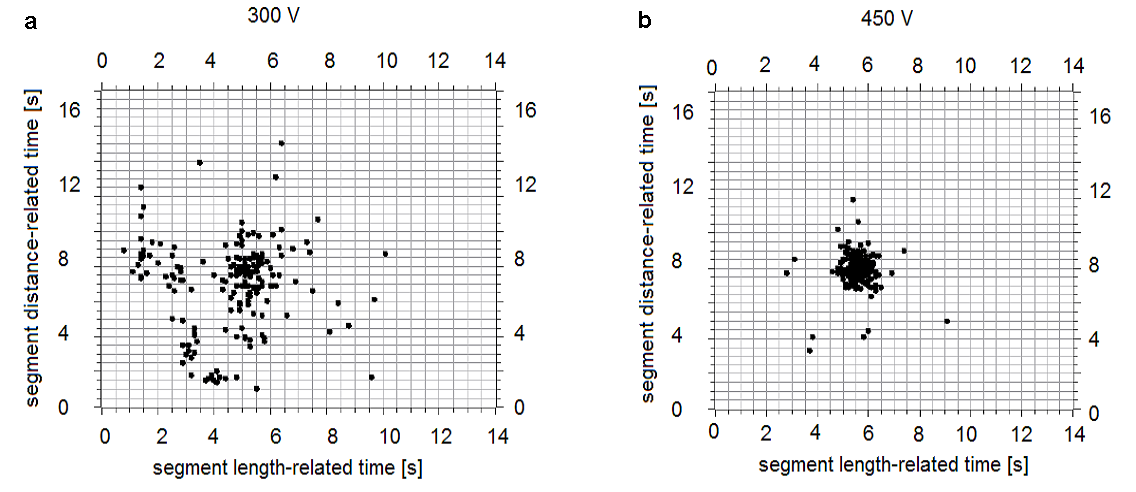

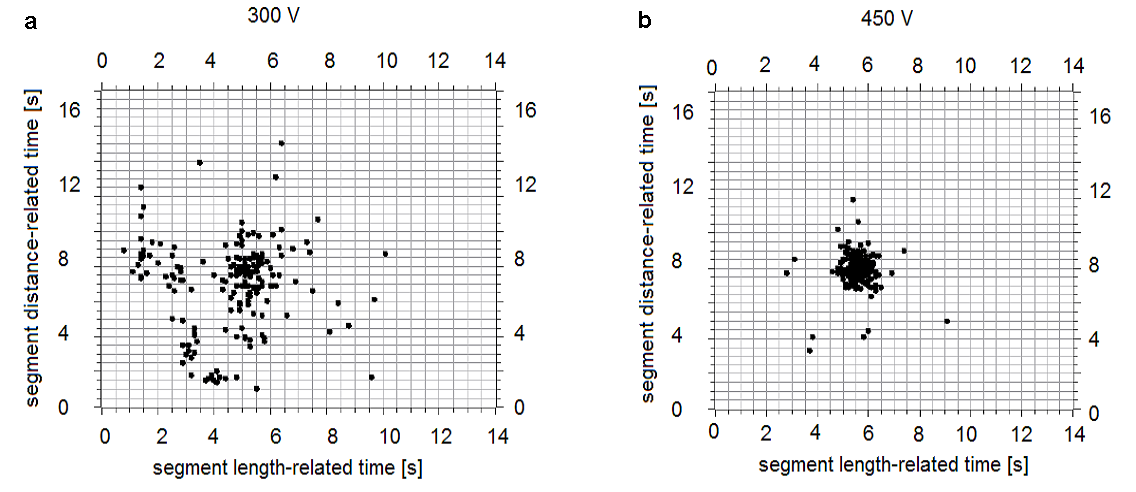


**Figure S6**: Sequence quality analysis: (**a**) scatter plot for segment distance and segment size-related time signals for an aqueous solution containing 20% of glycerol at 300 V, (**b**) scatter plot for segment distance- and segment size-related time signals for an aqueous solution containing 20% of glycerol at 450 V.
